# Alzheimer's Pathogenesis and Its Link to the Mitochondrion

**DOI:** 10.1155/2015/803942

**Published:** 2015-04-20

**Authors:** C. Simoncini, D. Orsucci, E. Caldarazzo Ienco, G. Siciliano, U. Bonuccelli, M. Mancuso

**Affiliations:** Department of Experimental and Clinical Medicine, Neurological Clinic, University of Pisa, Via Roma 67, 56126 Pisa, Italy

## Abstract

Alzheimer's disease (AD) is the most common form of dementia in the elderly. This neurodegenerative disorder is clinically characterized by impairment of cognitive functions and changes in behaviour and personality. The pathogenesis of AD is still unclear. Recent evidence supports some role of mitochondria dysfunction and oxidative stress in the development of the neurodegenerative process. In this review, we discuss the role of mitochondrial dysfunction in AD, focusing on the mechanisms that lead to mitochondrial impairment, oxidative stress, and neurodegeneration, a “vicious circle” that ends in dementia.

## 1. Alzheimer's Disease and Mild Cognitive Impairment

Alzheimer's disease (AD) is a degenerative brain disorder separately described in 1906 and 1910 by Alzheimer and Perusini [1–3]. AD is the most common form of dementia, affecting over 24 million of people worldwide, with 4.6 million new cases per year [4]. Usually, at the time of onset, age is between 60 and 70 years. The disease is clinically characterized by a chronic and disabling memory impairment in association with at least one other cognitive feature, resulting in a reduced competence to judge or to reflect. Cognitive functions that can be involved include the verbal and nonverbal memory, language, orientation, constructional abilities, abstract thinking, ability to solve problems, or praxis. There are frequently changes in behaviour and personality. Usually, death is caused by infections, pulmonary embolism, malnutrition, or heart disease. A definite diagnosis is only possible postmortem with autopsy [5]. The most common pathological features of AD include selective pyramidal neuronal death, accumulation of intraneuronal and extracellular fibrils, neurofibrillary tangles (NFT), and senile plaques [6, 7]. Senile plaques are filamentous aggregates of amyloid-beta peptide (A*β*) [8], whereas the main component of neurofibrillary tangles is the microtubule-associated protein tau [9]. These pathological deposits can be found during autopsy. In vivo, these aggregates may be detected among AD patients by imaging techniques such as positron emission tomography (PET) with Pittsburg compound B for amyloid [10] or 18F-THK23 for Tau aggregates [11], although further clinical studies are still needed to define the correct assay.

Mild cognitive impairment (MCI) is an intermediate stage between normal cognitive function and dementia [12], with deficits in at least one cognitive domain [13]. There are two forms of MCI: the amnestic MCI (memory impairment) and the nonamnestic MCI (multiple-MCI) with impairment in cognitive domains other than memory (e.g., frontal/executive, language, attention, or visuospatial skills) [14].

Currently, the pathogenesis of AD and the mechanisms leading to neuronal death have not been fully clarified. AD may be familial (FAD) and sporadic (SAD).

FAD represents a minority of AD cases, with autosomal dominant inheritance. Mutations in three known genes can cause FAD. These genes encode for amyloid precursor protein (APP) and presenilin-1 and presenilin-2 (PS1 and PS2) [15]. To date, the beta-amyloid cascade hypothesis remains the main pathogenetic model of FAD [16]. The A*β* peptide is the result of a regulated intramembrane proteolysis of APP by the sequential cleavage by *β*- and *γ*-secretases [17, 18]. A*β* plaques may be the cause of neurotoxicity, loss of synapses, and, ultimately, neuronal death [19, 20]. The exact mechanisms of the neurotoxicity of A*β* are still unknown. Several evidence suggest that A*β* exerts its toxicity intracellularly [21, 22]. Neurotoxicity of amyloid might play an important role in cell death by a cascade of events resulting in reactive oxygen species generation and oxidative stress [23]. Since mitochondria are the main site of free radical production [24], their involvement in neuronal degeneration has been hypothesized.

The main risk factor for SAD is aging [25]. Probably, SAD is the result of a complex interplay between acquired and genetic factors [26].

## 2. Positron Emission Tomography and Energy Metabolism in AD

In AAD, the cerebral metabolic rate for glucose is reduced in temporoparietal cortices; fluorodeoxyglucose positron emission tomography (FDG PET) can measure this reduction. This feature appears to relate to the clinical disabilities of AD patients and can also precede the clinical symptoms by decades [27]. Reduced cerebral metabolism preceded both the neuropsychological impairment and the cortical atrophy detected with conventional neuroimaging [28].

In agreement with these studies, decreased glucose utilization in skin fibroblasts of AD patients has been also observed [29]. Mitochondria are membrane-enclosed cytoplasmatic organelles, defined as “the powerhouse of all cells”; mitochondrial dysfunction will inevitably impair energy metabolism. In brain, the oxidation of glucose occurs through the tricarboxylic acid (TCA) cycle (the Krebs' cycle) in the mitochondria. Acetyl CoA, the metabolite necessary to initiate the TCA cycle, is obtained by the oxidative decarboxylation of pyruvate, the product of glycolysis, through the pyruvate dehydrogenase complex (PDHC), which includes eight different enzymes. Deficiency in the two key enzymes of rate limiting step of the TCA cycle, PDHC and *α*-ketoglutarate dehydrogenase complex (KGDHC), can explain the observed defects in glucose metabolism in the AD brains [30, 31]. In addition to reduced rate of glucose, other alterations in AD brains include decreased activity of isocitrate dehydrogenase and increased activity of succinate dehydrogenase and malate dehydrogenase [31]. All the changes observed in TCA cycle activities correlated with clinical state, suggesting that they could lead to diminished brain metabolism resulting in the decline in brain function [31].

Liang and coworkers, in a recent transcriptomic study, examined the expression of 80 metabolically relevant nuclear genes in brains of AD patients, comparing them to normal controls [32]. This study showed a significantly lower expression of the nuclear genes encoding subunits of the mitochondrial electron transport chain (ETC) in posterior cingulate cortex, in the middle temporal gyrus, in hippocampal CA1 cells, in entorhinal cortex, and in other brain areas of AD patients [32]. Western blots confirmed underexpression of the ETC subunits [32]. Thus, metabolic abnormalities in FDG PET studies of AD are associated with reduced neuronal expression of nuclear genes encoding subunits of the mitochondrial ETC.

## 3. Mitochondria and the Mitochondrial DNA

Mitochondria range from 0.5 to 10 micrometers (*μ*m) in diameter and have developed from a symbiotic relationship established 1.5 billion years ago between primitive aerobic bacteria with a single-cell anaerobic organism [33]. Mitochondria are ubiquitous in eukaryotes. They are composed of a smooth outer membrane surrounding an inner membrane of significantly larger surface area that, in turn, surrounds a protein-rich core, the matrix. They contain 2 to 10 molecules of DNA, the mitochondrial DNA (mtDNA) [34]. Their number per cell ranges from zero in erythrocytes to several thousands in striated muscle cells. Their main function is to support aerobic respiration and to provide energy as adenosine triphosphate (ATP), by means of the electron transport chain (ETC), which is needed for oxidative phosphorylation that provides the most efficient energetic outcome in terms of ATP production for cell. The ETC consists of four multimeric protein complexes (complexes I to IV) located in the inner mitochondrial membrane together with complex V [34]. The ETC also requires cytochrome c (cyt c) and a small electron carrier, coenzyme Q10 (CoQ10 or ubiquinone). Electrons are transported along the complexes to molecular oxygen (O_2_), finally producing water. At the same time, protons are pumped across the mitochondrial inner membrane, from the matrix to the intermembrane space, by the complexes I, III, and IV. This process creates an electrochemical proton gradient. ATP is produced by the influx of these protons back through the complex V, or ATP synthase (the “rotary motor”) [35].

Mitochondria are key regulators of cell survival and death. Dysfunction of mitochondrial energy metabolism leads to reduced ATP production, impaired calcium buffering, and increased generation of reactive oxygen species (ROS) [36]. Human mtDNA is a circular, double-stranded molecule of 16569 base pairs. It contains 37 genes, including 13 protein-encoding genes, 22 transfer RNA genes, and two ribosomal RNA genes. All 13 protein-encoding genes are components of the mitochondrial respiratory chain, which is located in the inner membrane [34].

## 4. Mitochondria and Oxidative Stress

Oxidative stress describes the condition in which cellular antioxidant defences are insufficient to keep ROS levels below a toxic threshold [37]. The accumulation of ROS damages several biomolecules, including lipids, proteins, and nucleic acids. The cells possess a complex network of defence mechanisms to neutralize excessive accumulation of ROS. Some tissues, especially the brain, are mostly vulnerable to oxidative stress because of their elevated consumption of oxygen and the consequent generation of large amounts of ROS. Moreover, compared to other tissues, the brain, from one hand, has a lower activity of antioxidant enzymes such as glutathione peroxidase and catalase and, to the other hand, it contains elevated concentrations of polyunsaturated fatty acids that are highly susceptible to lipid peroxidation [38]. Mitochondria generate ATP as electrons flow through mitochondrial complexes I to IV of the electron transport chain, from donors with lower redox potential to acceptors with higher redox potential [39]. The final acceptor is oxygen that is reduced to water. The generated energy drives the phosphorylation of ADP to ATP by the mitochondrial complex V (or ATP-synthase). Although, the transport of the electrons through mitochondrial complexes is an efficient process, ROS may be produced and this amount is higher in damaged and aged mitochondria. In several neurodegenerative diseases, mitochondrial dysfunction is one of the earliest and prominent features [40–42], leading to increased oxidative stress neurons [43]. Additionally, mitochondria are a target for ROS, causing the oxidation of their components such as mtDNA, lipids, and proteins in a vicious circle [44].

## 5. Mitochondrial Oxidative Stress and AD

The possible relationship between oxidative stress and AD originally derived from the free radical hypothesis of aging, suggesting that age-related accumulation of ROS could be responsible of damage to major cell components [36]. ROS increase is consequent to mitochondrial dysfunction linked to aging and mitochondrial dysfunction results to be accelerated in AD patients [45]. As demonstrated by several studies, mitochondrial dysfunction and increased oxygen species generation play an important role in AD [25, 36]. Increased oxygen species production and deficient mitochondrial dynamic balance have been suggested to be both reason and consequence of AD related pathology. This event may correlate with the presence of dysfunctional mitochondria, resulting in reactive oxygen species generation (ROS) [46]. Mitochondria generate cell energy as electrons flow through electron transport chain. Although this mechanism is an efficient process, some ROS may be produced and dysfunctional mitochondria can generate high levels of ROS with a toxic effect for cells with a long life span and a deficiency in antioxidant defences, including cerebral cells [43]. Furthermore, mitochondria may be themselves damaged by ROS, causing a “vicious circle,” resulting in additional mitochondrial impairment. Aging-related oxidative damage may be a significant risk factor for AD [25]. CNS functions heavily depend on efficient mitochondrial activity, since brain tissue requires high energy provision. However, it is unclear whether mitochondrial impairment and oxidative stress are actually involved at the onset or in the progression of the neurodegenerative process.

Trials with antioxidant agents such as vitamin E have shown controversial results; Sano and coworkers [47] demonstrated that vitamin E slows the progression of disease in moderately severe AD while Petersen et al. did not demonstrated a significant benefit of this antioxidant agent in patients with MCI [48]. Dumont and colleagues examined the role of Coenzyme Q10 in the Tg19959 transgenic mouse model of AD that overexpress A*β*PP, demonstrating a positive effect of this factor both on pathology and behaviour in this model [49].

## 6. “Cybrid” Models of AD

Cybrid cells are generated by mixing the contents of nonnucleated cells or cytoplasts, with nucleated cells [50]. A particular case of cybrid cells is formed by the fusion of cells completely private of mitochondrial DNA and containing only nuclear DNA (nDNA), the so-called cell rho-zero, with cytoplasts containing only mitochondrial DNA (mtDNA). It is important to note that cybrid cell lines are not created through the transfer of isolated mtDNA, but rather through the transfer of whole mitochondria to *ρ*0 cells. All transferred cytoplast components that cannot be perpetuated independently of the host cell nucleus degrade over time and dilute over the course of repeated cell divisions. Therefore, any transferred cytoplast component that cannot perpetuate itself independent of the cell nucleus should not have a sustainable molecular or biochemical effect. Theoretically, the only self-perpetuating component is the mtDNA. These cybrid cells can be a model to study central nervous disease, such as AD. The first studies of cybrid cell lines containing mtDNA from AD patients were published in 1997 [50]. These studies demonstrated that, in AD cybrids, cytochrome c oxidase (COX), the complex IV of the respiratory chain lower than in the corresponding control cybrid group [51, 52]. COX activity reduction was associated with reduced ATP in the cell and increased ROS production. Finally, AD cybrids contained more intracellular APP products than the control cybrids [53–56].

## 7. Is There a Role for the Mitochondrial DNA in AD?

Even if cybrid studies have suggested mtDNA contribution to mitochondrial dysfunction in AD, studies attempting to identify mtDNA mutations in the brains of AD patients had limited success. For example, Elson et al. [57] sequenced the complete coding regions of 145 autoptic AD brain samples and 128 controls. They observed that the overall numbers of mtDNA nucleotide substitutions were the same for the AD and for the control sequences [57]. So far, no causative mtDNA mutations have been linked to AD. Chagnon and coworkers [58] reported that haplogroup T is underrepresented in AD patients, while haplogroup J seems to be overrepresented. By studying a sample of Italian subjects, Carrieri et al. [59] showed that haplogroups K and U were present at a lower frequency in AD patients, who were apolipoprotein (Apo) E4 carriers than in noncarriers, while in controls there was no association between the E4 allele and mtDNA haplogroups. This evidence suggests that K and U may act by neutralising the effect of the major AD risk factor, the E4 allele [59]. Another report demonstrated that males classified as haplogroup U had a significantly increased risk of AD, while females had a significantly decreased risk with the same U haplogroup [60]. Two additional studies, including only neuropathologically proven cases of AD in European patients indicated that mtDNA haplogroups were not associated with AD, either individually or by grouping together closely related haplogroups [57, 61]. Another study did not suggest an aetiological role of haplogroup-associated polymorphisms [62]. In conclusion, although the suggestions of a possible relation between haplogroups K and U and risk of AD in Caucasian population, this field of research is still to be fully explored. To date, mtDNA haplogroups do not seem to play a major role in AD pathogenesis [63].

## 8. APP, A***β***, and Mitochondria

The human* APP* gene, first identified in 1987, is located on chromosome 21. Several evidences suggest that mutations in the* APP* gene are correlated to FAD and a related condition, cerebral amyloid angiopathy [15, 64]. The cleavage of APP produces two major forms of A*β* peptides, A*β*40 (the prevalent isoform), and the more pathogenic and less expressed A*β*42. A number of functions have been attributed to APP, but its function is not fully understood to date.

Li et al. have shown a casual relationship between oxidative stress and AD plaque pathology using APP mutant MnSOD+/−heterozygous knockout mice. In addition, Esposito and coworkers showed that partial reduction in the main mitochondrial superoxide scavenger Sod2 accelerates the onset of hAPP/A*β*-dependent behavioural abnormalities and worsens a range of AD-related molecular and pathological alterations [65, 66].

However, others preclinical models with transfected cells and transgenic mice have shown that overexpression of APP produces mitochondrial dysfunction through the block of the mitochondrial protein import machinery [67]. Oxidative stress may facilitate the expression of a protein involved in the generation of A*β* and the beta-secretase (BACE 1) [68] and, moreover, may induces a pathogenic PS1 conformational change in neurons, increasing A*β*42/40 ratio, as shown by in vitro models [69]. *β*-amyloid protein (A*β*) interacts with the binding alcohol dehydrogenase protein (ABAD), a mitochondrial-matrix protein, causing mitochondrial oxidative damage and deficiency of the respiratory complexes [70, 71]. In view of the fact that A*β* is not produced locally in the mitochondrion [67], Hansson Petersen and coworkers investigated the mechanisms by which A*β* is taken up by mitochondria [72]. The most important system providing the translocation of A*β* precursors with mitochondrial target signals involves both the translocase of the outer membrane (TOM) and the translocase of the inner membrane (TIM). Targeting signals are first recognized by TOM receptors (Tom20, Tom22, and Tom70), and then traslocated by Tom40, the general import pore of TOM [73, 74]. Subsequently, A*β* precursors are directed to the matrix via the Tim23 complex [74]. In isolated rat mitochondria has been observed that A*β* is imported into mitochondria via the TOM complex [72]. With the preincubation of mitochondria with antibodies directed toward Tom20, Tom40, or Tom70, the import of A*β* is decreased [72]. The import into mitochondria was insensitive to the mitochondrial membrane potential dissipater valinomycin, indicating that it is independent of the membrane potential of the mitochondrion [72]. A*β* also has many other functions such as interaction with HtrA2/Omi, a proapoptotic serine protease released into the cytoplasm by mitochondria on apoptotic stimulation [75], inhibition of ketoglutarate dehydrogenase complex, and reduction of COX activity [76]. An important role of A*β* in modulating proteins involved in mitochondrial fission/fusion processes was recently suggested. Mitochondria are highly dynamic organelles, ranging from giant tubular networks to small round entities through rapid and reversible fission and fusion processes. In hippocampal tissues of AD patients, reduced levels GTPase dynamin-like protein 1 (DLP1), OPA1, Mfn1, and Mfn2 and increased levels of Fis1 have been found, suggesting impaired mitochondrial dynamics in favour of fission. A proper balance of fusion and fission proteins is fundamental for the correct distribution of the mitochondria in the cells [77]. This is particularly important for neurons that may have very long axons and also for the function of synapses, which are subcellular regions with high metabolic requirement [78].

## 9. Tau Protein and Mitochondria

Tau is a normal neuronal protein, which modulates microtubule-based functions. Multiple studies demonstrated that tau plays a crucial role in the pathogenesis of AD, given that in animal models of AD reducing tau levels attenuates neuronal dysfunction [79, 80], and in humans the extent of tau pathology correlates with cognitive impairment [81]. Neurofibrillary tangles (NFTs), insoluble fibrillar intraneuronal accumulations of modified tau protein, are important and define hallmarks of the disease. The main modification of tau in the NFTs is hyperphosphorylation; however, numerous modifications have been reported, including truncation, acetylation, and nitration [82, 83]. As mentioned above, reduced cell energy due to complexes I and IV inhibition promotes tau phosphorylation [84], suggesting that mitochondrial damage could play a role in facilitating tangles formation and thus neurodegeneration. The products of the toxic action of ROS may facilitate the self-assembly of the Tau protein into fibrillary polymers similar to the paired helical filaments present in the brain of AD patients [85]. On the other hand tau accumulation caused mitochondrial dysfunction in a mouse model of AD [86].

## 10. Vascular Factors and Mitochondrial Dysfunction

Pathological studies have shown reduced number of cerebral microvessels, decreased capillary diameters, and increased capillary densities in AD brains [87], with atrophy of smooth muscle cells in brain vessels and attenuation of capillary endothelium, resulting in the rupture of the vessel wall [88]. Atherosclerosis has also been observed in the circle of Willis and in large leptomeningeal vessels [89, 90].

Cerebral hypoperfusion may be a preclinical condition of AD [91]. The reduced cerebral blood flow (CBF) may play a critical role in the degenerative cascade leading to AD. Mitochondria in vulnerable cells show signs of damage during ischemia [92]. Chronic hypoperfusion can trigger mitochondrial dysfunction in vascular cells, which, in turn, will enhance both production and accumulation of ROS. When the ETC is inhibited, including when the regional CBF is reduced, the electrons are donated directly to oxygen to give an anion superoxide. Superoxide can react with nitric oxide (NO) to form peroxynitrite, which is severely damaging for DNA and RNA. NO, which acts as vasodilator, is reduced by increased levels of ROS. Thus, increased oxidative stress interferes with NO function and vascular relaxation. This scenario could play a role in the vascular abnormalities underlying metabolic defects in AD [92]. Moreover, increased levels of oxidative stress have been reported in AD brains [90]. Immunocytochemical studies revealed that the regional distribution of RNA oxidation in the brain was consistent with the selective neuronal vulnerability [80]. There were increased levels of 8-hydroxyguanosine (8-OHG) in the hippocampus and cerebral neocortex in AD, whereas no alteration in the 8-OHG levels was found in the cerebellum [93]. Oxidized RNAs were localized predominantly in neuronal and endothelial cells compared with the glia [94], and oxidized RNA in endothelial cells could have a key role in the degenerative cascade [90]. Aliyev and coworkers [95] described the ultrastructural features of vascular and mitochondrial alterations in vascular wall cells from human AD brain biopsies. As expected, there was a higher degree of A*β* deposition in the vascular walls in AD compared to age-matched controls [95]. Moreover, increased levels of 8-OHG and significantly more mitochondrial abnormalities were seen in the vascular endothelium and in the perivascular cells of the microvessels where atherosclerotic lesions occurred [96]. These features were absent in unaffected regions of AD or in age-matched control subjects [95], suggesting that vascular wall cells, and especially their mitochondria, may be a central target for oxidative damage before the development of AD pathology [90].

## 11. Conclusion

AD is the most common form of adult onset dementia. Its etiology is complex, and only a minority of cases are genetically inherited (FAD). The etiology of sporadic forms of AD (SAD) is still a controversial area. The relationship between mitochondrial pathology and APP and beta-amyloid metabolism is controversial as well. For instance, the cellular trafficking of APP and beta-amyloid are quite well understood but there is not a general consensus about their import in the mitochondria [97, 98]. There is still a strong need for a better understanding of these connections.

Multiple interactions among genetic and environmental factors appear to be causative of the sporadic forms. Accumulation of somatic mtDNA mutations accelerates normal aging, leads to oxidative damage, and causes energy failure, increased production of ROS and amyloid accumulation, which in a vicious manner reinforces the mitochondrion damage, the impairment of the mitochondrial respiration, and the accumulation of ROS. Endothelial mtDNA deletions themselves seem to contribute to the pathogenesis, but the exact mechanism of that is still unclear.

Most likely, the mtDNA does not play a primary role, and, therefore, it could be involved subsequently [90]. A recent, large study excluded any association between AD and genetic variation of mtDNA, and meta-analysis of the available data showed no evidence of an association with AD [96]. It will be important to develop a better understanding of the role of oxidative stress and mitochondrial energy metabolism, and its links with the amyloid hypothesis and the endothelial and vascular dysfunction observed in AD [90], since it may lead to the development of more effective treatment strategies for this devastating disorder.
